# P37 Rare case of hemophagocytic lymphohistiocytosis triggered by Covid-19 infection in lupus patient complicated by decompensated cardiomyopathy

**DOI:** 10.1093/rap/rkae117.068

**Published:** 2024-11-05

**Authors:** Ramadan Musa

**Affiliations:** City Hospital, Birmingham, United Kingdom

## Abstract

**Introduction:**

Covid-19 infections in immune-compromised patients can cause severe disease especially if the patient is anaemic, underweight, and immune-compromised with methotrexate therapy. Severe COVID-19 infection can trigger hyper-immune activation causing hemophagocytic lymphohistiocytosis (HLH) syndrome resulting in life-threatening multi-organ failure [1]. HLH can either be primary usually in children or secondary due to infections, malignancies, or autoimmune diseases like lupus [2]. Mild initial symptoms may deteriorate rapidly which is why early diagnosis is essential for survival [3]. Treatment must be started in case of suspicion based on a combination of clinical signs and blood tests 2004 HLH-criteria [4].

**Case description:**

We report a case of a 27-year-old female, physiotherapist originally from India, diagnosed initially with rheumatoid arthritis. On the 7^th^ of September 2023, she was asymptomatic on 15 mg of methotrexate weekly oral. On the 5th of October, she developed a dry cough and tiredness and tested positive for Covid-19, she was advised to stop methotrexate. After two weeks she tested negative for COVID-19 but the cough and tiredness persisted. On the 14th of November, she became unwell, developed a fever of 38.4, skin rash, continued cough, and shortness of breath. She was admitted to the Hospital with, BP 109/65, pulse 110/min, O2 Sat 98% on 21% oxygen, respiratory rate 24/min. Blood tests show pancytopenia Hb 74, WBC 1.3, neutrophil 0.79, platelet 58, ESR 105, ferritin 9468, and triglyceride 3.3. She looked pale, dehydrated, and had mucosal ulceration, weight 46.2kg, urine PCR 433.

The patient had a negative septic screen. Chest X-ray showed bilateral basal consolidation. She was started on IV fluid, IV tazocin, and given IV methylprednisolone 500 over 3 days, followed by oral prednisolone 40 mg daily, she was fed through an NG tube and had 3 units of blood transfusion. There was an initial improvement in the pancytopenia Hb 98, WBC 7.10, platelet 125, but she suddenly developed progressive shortness of breath, BP 90/58, and tachycardia 165/min, CT-PA excluded PE. Echo shows a drop in LVEF <15%, troponin I 33.7. The patient transferred to ITU and started inotrope which improved BP to 105/75. The patient had their first dose of RIT on the 15th of December, followed by Cyclo 500 mg on the 18th of December, after 4 days she showed remarkable recovery blood markers and general condition, and she had 3 cycles of cyclo, she was discharged on 23rd of January 2024, repeat ECHO showed LVEF 60 - 65%.

**Discussion:**

Covid-19 induced HLH in under-weight immune compromised lupus patient has not been reported before. In our case, she was immune-compromised with methotrexate therapy, in addition, she was under-weight (BMI 16.9), and anaemic (low serum iron 4.8, and had low vitamin B12 (19)). The infection triggers hyper-immune response resulting in secondary HLH. Diagnosis of HLH in our patient was difficult because she was anaemic with sepsis and both can cause pancytopenia. Many signs made a diagnosis of lupus more likely in our patient than rheumatoid arthritis like ANA positive, anti-SM positive, scaring alopecia, malar rash, mucosal ulceration, and pancytopenia.

In the management of HLH, early identification and treatment of the underlying triggering factor is essential. However, if the patient is unstable, then treatment with IV methyl-prednisolone 500 mg X3 should be started without delay. Early admission to ITU facilitates monitoring and early detection of complications and helps initiate supportive therapy if needed, as our patient after initial improvement deteriorates suddenly due to complications of decompensated cardiomyopathy with drops in LVEF <15%, raised troponin I and drop in blood pressure, she had a brief period of hemofiltration and Inotropic therapy with dobutamine. Further treatment depends on the underlying cause. In our patient, there were high suspicions of lupus as an underlying trigger factor, and treatment with rituximab & cyclo (euro-lupus) was initiated with good effect as BP improved and inotropic therapy was withdrawn. She wined off prednisolone slowly and started on mycophenolate 1 gm bd oral before discharge.

**Key learning points:**

• The outcome of HLH depends on early recognition of the clinical triad of fever >38, pancytopenia, hyperferritinemia, and hypertriglyceridemia, which form part of the 2004 HLH criteria

• The disease usually progresses very quickly to multi-organ failure and has a high mortality rate of 40%, our patient deteriorate rapidly from flu-like symptoms to becoming very unwell.

• Early initiation of treatment is crucial for survival, and with the earliest suspicion of HLH, steroids should be started without delay, in our patient initially we started methylprednisolone 500 mg IV X3, followed by IV hydrocortisone 50 mg bd as the patient was unstable.

• Admission to ITU should be considered early to help monitor vital organs and provide supportive therapy if necessary, in our patient admission to ITU helped initiate inotropic therapy early to support the heart when the patient develops decompensated cardiomyopathy and enable to start hemofiltration when the patient develops academia.

• The ultimate goal of therapy is to suppress the uncontrolled immune activation process with appropriate immunosuppression therapy depending on the condition of the patient and the underlying cause of HLH, in our patient the underlying diagnosis was lupus flare, and treatment with rituximab followed by cyclo was appropriate as the patient was unstable and cyclo in combination with rituximab work quicker. Regarding consent for treatment the patient was drowsy and unwell with LVEF <15%, so we spoke to her parent and fiancé about the side effects of the cyclo and rituximab and they agreed to treatment. The patient made a quick recovery within 4 days she restored her blood pressure and inotropic therapy was stopped, within a week she was able to stand and sit in the chair. The repeated echo and MRI heart show normal heart function with LVEF 60 to 65%.

